# Multidisciplinary paper on patient blood management in cardiothoracic surgery in the UK: perspectives on practice during COVID-19

**DOI:** 10.1186/s13019-023-02195-4

**Published:** 2023-04-01

**Authors:** Nawwar Al-Attar, Jullien Gaer, Vincenzo Giordano, Emma Harris, Alan Kirk, Mahmoud Loubani, Patrick Meybohm, Rana Sayeed, Ulrich Stock, Jennifer Travers, Becky Whiteman

**Affiliations:** 1 Golden Jubilee National Hospital, University of Glasgow, Agamemnon Street, Clydebank, Glasgow, G81 4DY Scotland, UK; 2 Royal Brompton and Harefield NHS Foundation Trust, London, UK; 3 Department of Cardiothoracic Surgery, Royal Infirmary Edinburgh, Edinburgh, UK; 4 Royal Brompton and Harefield NHS Foundation Trust, London, UK; 5 Department of Thoracic Surgery, Golden Jubilee National Hospital, Glasgow, UK; 6 Hull University Teaching Hospitals NHS Trust, Hull, UK; 7 Department of Anaesthesiology, Intensive Care, Emergency and Pain Medicine, University Hospital Wuerzburg, Würzburg, Germany; 8 Oxford University Hospitals NHS Foundation Trust, Oxford, UK; 9 Royal Brompton and Harefield NHS Foundation Trust, London, UK; 10 West of Scotland Cancer Centre, Golden Jubilee National Hospital, Glasgow, UK; 11 Cluster Medical Manager Advanced Surgery – UKI and Nordics Worldwide Medical, Baxter Healthcare Limited, Berkshire, UK

**Keywords:** Blood management, Bleeding, Cardiothoracic surgery, COVID-19, Haemostats

## Abstract

The coronavirus (COVID-19) pandemic disrupted all surgical specialties significantly and exerted additional pressures on the overburdened United Kingdom (UK) National Health Service. Healthcare professionals in the UK have had to adapt their practice. In particular, surgeons have faced organisational and technical challenges treating patients who carried higher risks, were more urgent and could not wait for prehabilitation or optimisation before their intervention. Furthermore, there were implications for blood transfusion with uncertain patterns of demand, reductions in donations and loss of crucial staff because of sickness and public health restrictions. Previous guidelines have attempted to address the control of bleeding and its consequences after cardiothoracic surgery, but there have been no targeted recommendations in light of the recent COVID-19 challenges. In this context, and with a focus on the perioperative period, an expert multidisciplinary Task Force reviewed the impact of bleeding in cardiothoracic surgery, explored different aspects of patient blood management with a focus on the use of haemostats as adjuncts to conventional surgical techniques and proposed best practice recommendations in the UK.

## Introduction and rationale

Bleeding is one of the main complications of cardiac surgery. Herein, a multidisciplinary Task Force outlines the principles of patient blood management in cardiothoracic surgery with a focus on perioperative assessment and communication tools, and proposes recommendations for selection of surgical haemostats particularly in the context of the coronavirus (COVID-19) pandemic and its fallout in the United Kingdom (UK), both in terms of disruption of supply of blood products and decline in cardiac procedural activity with a deficit in excess of 45,000 procedures [1, 2]. Although adjusted 30-day mortality was similar in the pre-COVID and COVID time periods, further studies demonstrated a significant increase in operative mortality with a more complicated postoperative course particularly in terms of postoperative bleeding [3]. While several medical societies have highlighted the essential role of patient blood management (PBM) in the management of pandemics and encouraged the implementation of the principles of PBM [4], this work is principally dedicated to practice of cardiothoracic surgery.

## Impact of bleeding

Despite optimal surgical management, post-operative bleeding in cardiac surgery patients occurs at a frequency of 10%; half of these patients require a return to theatre for surgical haemostasis and half experience diffuse microvascular bleeding [5]. Microvascular bleeding is most likely secondary to the multiple effects of the cardiopulmonary bypass (CPB) circuit on coagulation proteins and platelets [6]. These bleeding complications are associated with increased length of hospital stay, intensive care unit support, and inferior clinical outcomes overall [7]. In-hospital mortality is substantially higher in patients who experience bleeding complications [8, 9]. Additionally, a dose-dependent inverse relationship can be demonstrated between increasing red blood cell transfusion and mortality.

Donated blood is an expensive and precious resource, an important consideration in an economically challenged health care system. The recent COVID-19 pandemic has highlighted the many challenges to ensuring adequate blood supply to meet clinical demand. Bleeding following cardiac surgery accounts for around 10% of national blood supply usage [5]. Attention has focussed on improving blood product management in bleeding patients. Timely and optimal blood product replacement is still an essential part of blood management in these patients, and may be lifesaving. Numerous publications have confirmed improved outcomes by adopting an agreed institutional algorithm, with pre-defined thresholds for transfusion of blood products or factor concentrates, in the management of post-operative cardiac bleeding based on laboratory coagulation assays or point of care tests [10–12]. This approach is favoured in preference to individual decision making and has been demonstrated across several studies to reduce overall usage of allogeneic blood components, hence reducing the number of donor exposures [10–12]. There is little evidence to endorse the use of empirical blood product support in stable, non-bleeding post-surgery patients following primary haemostasis [13].

Despite universal leucodepletion of blood products, an immunomodulatory effect is still likely to occur, along with other recognised hazards of transfusion including circulatory overload, transfusion-related lung injury, transfusion reactions, and transfusion-transmitted infection [13]. These potential hazards support adoption of a restrictive blood transfusion policy in the management of post-operative anaemia, where the level of haematocrit may be a poor indicator of tissue oxygenation [9]. Blood conservation policies should be implemented and enforced by individual centres, incorporating them into their hospital governance systems. In a prospective observational cohort study, postoperative bleeding was significantly higher during the COVID-19 pandemic (10.5%) compared to a historical cohort (2.9%), *p* = 0.003 [3].

## Preoperative management

### Coagulation screening

Routine coagulation testing remains standard practice as part of the surgical pre-assessment at many institutions to identify any abnormality that might increase the risk of perioperative bleeding. However, a normal coagulation screen can be mistakenly reassuring. A coagulation screen includes a prothrombin time, an activated partial thromboplastin time (APTT), and a thrombin time. Ninety-five percent of the adult population is expected to lie within the normal ranges provided by the testing laboratory. The results of these tests may not accurately reflect the complex interactions of haemostasis in vivo, between vasculature, clotting factors, platelets and fibrinogen, and may not therefore correlate well with the actual perioperative bleeding risk [14]. Prolongation of these tests may indicate a deficiency of one or more coagulation factors requiring specialist haematological advice and pre-operative management. Laboratory tests may also be prolonged by pre-analytic variables and phospholipid antibodies, neither of which cause bleeding [15].

In advance of a surgical procedure, it is often feared that without undertaking a coagulation screen a bleeding disorder may be missed. The majority of patients with a moderate to severe congenital bleeding tendency will have exhibited bleeding following haemostatic challenges early in life, and/or have a positive family history, meaning that they are already aware of their underlying condition.

It is widely accepted that undertaking a structured personal bleeding history, for example using the International Society on Thrombosis and Haemostasis validated bleeding assessment tool (ISTH-BAT), combined with exploring a family history of bleeding and information on concomitant use of antiplatelet and antithrombotic medications, is an effective way of identifying individuals with an increased bleeding tendency [16]. Selective laboratory testing can then be undertaken in those individuals identified with a high bleeding risk. Furthermore, in mild to moderate von Willebrand disease, one of the more common bleeding disorders, the coagulation screen may be normal, and the disorder may be missed if a detailed personal and family history is not taken. The limiting factor of this approach may be those individuals who have never undergone a significant operative challenge and, as such, not had a challenge of their haemostatic system.

Within the UK, the British Society for Haematology caution against the practice of unselected coagulation screening and advise selective screening in those with a positive personal or family bleeding history, patients on anticoagulants and those patients with a relevant medical history including cancer, liver disease, sepsis and disseminated intravascular coagulation [17]. The National Institute for Health and Care Excellence in the UK also recommends a similar approach [18]. There is no international consensus. US guidance suggests extra caution and recommends coagulation screening in higher-risk surgical cases. The Italian Society for Thrombosis and Haemostasis continues to recommend routine coagulation testing for all due to the lack of robust randomised controlled clinical trials [19, 20].

However, in higher risk surgery such as cardiac surgery, undertaking a coagulation screen at baseline may still be recommended for those patients requiring CPB, when heparin anticoagulation monitoring can prove challenging if there is baseline coagulation abnormality. For example, factor XII (Hageman factor) deficiency or lupus anticoagulant will both lead to isolated APTT prolongation without a clinical bleeding risk, but will also prolong the baseline activated coagulation time (ACT). In these cases, ACT monitoring during bypass may not accurately measure the effect of heparin.

We undertake coagulation screening in patients who will require CPB or extra corporeal membrane oxygenation circuits. This allows us to offer an alternative heparin monitoring strategy using the anti-factor Xa assay for those patients with baseline APTT abnormalities. The anti Xa test is not affected by variables interfering with the ACT test. However, this assay may not be readily available with a fast enough turnaround time in all institutions.

### Anaesthetic considerations

Pre-operative anaemia is common in cardiac surgery with a prevalence of between 10 and 50% (depending on definition) [21]. Pre-operative anaemia is mostly the result of inadequate erythropoiesis due to iron deficiency, malnutrition, malabsorption, inflammation, bone marrow disorders, or chronic blood loss [22]. Importantly, anaemic patients need to be identified, as early as possible, by the cardiologist, surgeon or anaesthetist [23]. The International Consensus Conference on PBM 2018, defined the current status of the PBM evidence base for clinical practice and research purposes, and established four clinical and three research recommendations for pre-operative anaemia, including the strong recommendation to detect and manage anaemia sufficiently early before major elective surgery [24].

Assessment of full blood count, ferritin, transferrin saturation, and the soluble transferrin receptor are recommended to identify any iron deficiency with or without anaemia [22]. Anaemia is often found in cardiac surgery patients due to infection or chronic kidney disease resulting in functional iron deficiency. After iron supplementation, haemoglobin concentrations rise approximately 1–2 g/dL within 2–4 weeks. A multicentre observational study by the UK Association of Cardiothoracic Anaesthesia and Critical Care Research Network found that the development of an intravenous iron pathway is feasible, but appears limited to selected high-risk cardiac patients in routine NHS practice. Patients with anaemia who received intravenous iron supplementation before surgery were at higher surgical risk, were more likely to have a known previous history of iron deficiency or anaemia, had a higher rate of chronic kidney disease and were slightly more anaemic than those who did not receive any iron supplementation [25]. A recent meta-analysis including four randomised controlled trials and seven observational studies suggested that administration of intravenous iron reduced the number of patients transfused and improved post-operative morbidity in adult cardiac surgery patients with pre-operative anaemia or iron deficiency [26].

An ongoing multicentre interventional trial (NCT02632760) with 1000 participants is expected to provide definitive answers about the effects of intravenous iron before cardiac surgery with the primary outcome measure as the number of days alive and out of hospital up to 90 days post operatively. A recent trial of 505 patients with iron deficiency or anaemia undergoing cardiac surgery investigated the effects of ultra short-term treatment using a combination of iron, erythropoietin, vitamin B12, and folic acid. During the first 7 days, significant reductions were observed in red cell concentrate transfusions, as well as significant increases in haemoglobin concentration, reticulocyte count, and haemoglobin content of reticulocytes (RET-He) [27]. Thus, iron supplementation, especially in combination with erythropoietin is recommended when the anaemia of chronic disease is accompanied by iron deficiency with complete depletion of iron stores [28]. Importantly, diagnosing and (if indicated) correcting pre-operative anaemia should be mandatory ahead of planned cardiac surgeries. In urgent cases, patients should still receive ultrashort-term pre-operative treatment [27].

## Operative management of bleeding

Although discussion of a Major Haemorrhage Protocol (MHP), with pre-defined transfusion thresholds, is out with the scope of this review, it is important that staff involved in cardiothoracic surgery are trained to recognize major blood loss early and be familiar with the institutional MHP. Staff must understand when to activate or deactivate this. The MHP should be tailored to cardiothoracic surgery and take into account the different methods of assessing coagulation. As highlighted in the recent guideline by Stanworth and colleagues, whether standard laboratory testing or near patient viscoelastic tests (TEG and Rotem) are used, there should be pre-defined thresholds and repeated testing employed, with comparison between longitudinal tests being more useful than a standalone result to help guide plasma and component use [10, 11, 29].

### Cardiopulmonary bypass/extracorporeal circulation considerations

Cardiopulmonary bypass is essential to perform most cardiac operations. The heart is connected to a roller or centrifugal pump via cannula in the right atrium and aorta. The prime fluid in the bypass circuit is usually made up of clear fluids and, therefore, results in haemodilution. Haemodilution on CPB increases the possibility of needing red cell transfusion during or after the procedure and this risk is related to the volume of the prime used [30]. There are several techniques to minimise haemodilution, including prime displacement (autologous priming), when the prime fluid is displaced by the patient’s own blood as the bypass is initiated, and the use of minimised extracorporeal circulation circuits. In both techniques, reduction of the clear fluid prime volume mixed with the patient’s blood minimises haemodilution and maintains a higher haemoglobin concentration during and after CPB leading to a lower requirement for red cell transfusion. Prime displacement (autologous priming) may be antegrade or retrograde and both techniques have been demonstrated to reduce blood transfusion following CPB [31–33]. There is normally a fall in blood pressure during prime displacement leading to concerns about impaired organ perfusion and increased vasopressor support during volume reduction. However, the effect on blood pressure has been shown to be transitory and results in no long-term impact on patients. Although cerebral oxygen saturation has been found to fall on induction of CPB using prime displacement, this is also evident during conventional CPB without prime displacement with no clinically significant consequences [31].

Minimised extracorporeal circulation (MECC) technology has evolved from the original type I to the most recent type IV circuits; it incorporates all of the recent advances in perfusion science, with a closed circuit and much-reduced priming volume and has been shown to maintain haematocrit and reduce blood transfusion requirement compared with conventional bypass, but not over prime displacement [31, 34, 35]. Prime displacement has the advantage of being applicable in all cardiac operations although MECC use has been extended from coronary artery bypass surgery to aortic valve surgery, and even mitral valve surgery. Another strategy is to use a patient-specific CPB circuit related to body surface area [36]. However, although this approach resulted in a significant reduction in on-bypass haemodilution and increased post-operative haematocrit levels, it had no impact on transfusion requirement.

### Cell salvage

Currently, even though there is no evidence to confirm COVID-19 transmission through blood transfusion, there is a general unwillingness to donate blood, which may result from fear and a desire to maintain social distancing [37]. Unfortunately, this has not decreased the requirement for blood transfusion, especially during cardiac operations. Moreover, with the rising cost of allogenic blood administration, the use of cell salvage has surged as a valuable strategy in numerous types of invasive procedures [38].

In general, a cell salvage machine is used to prevent red blood cells wastage. The patient’s shed blood aspirated from the surgical field is filtered, washed, and processed to ensure the removal of impurities, including coagulation and inflammatory markers, and the red cells are returned to the patient [39, 40]. According to literature the usage of cell saver machines has neither multiplied nor diminished, but with a shortage in blood supply, cell salvage is the optimum option to reduce transfusion rate and associated potential complications, such as infection [38]. The cost of equipment however needs to be weighed against the price of blood in countries where this is charged. It could be argued that in comparison to the cost and risk of receiving allogeneic red cells, it is safer and more cost-effective. In addition, blood loss can never be predicted. Cell salvage is however not entirely without problems: removing platelets and haemostatic blood factors especially if a large volume of blood is processed, might increase the risk of post-operative bleeding and the requirement for allogenic blood products [41]. Cell salvage remains an essential tool in cardiac surgery and is even more relevant in the COVID-19 pandemic due to the challenge for blood availability and supply.

### The validated intraoperative bleeding scale in practice: a communication tool

**Fig. 1:**
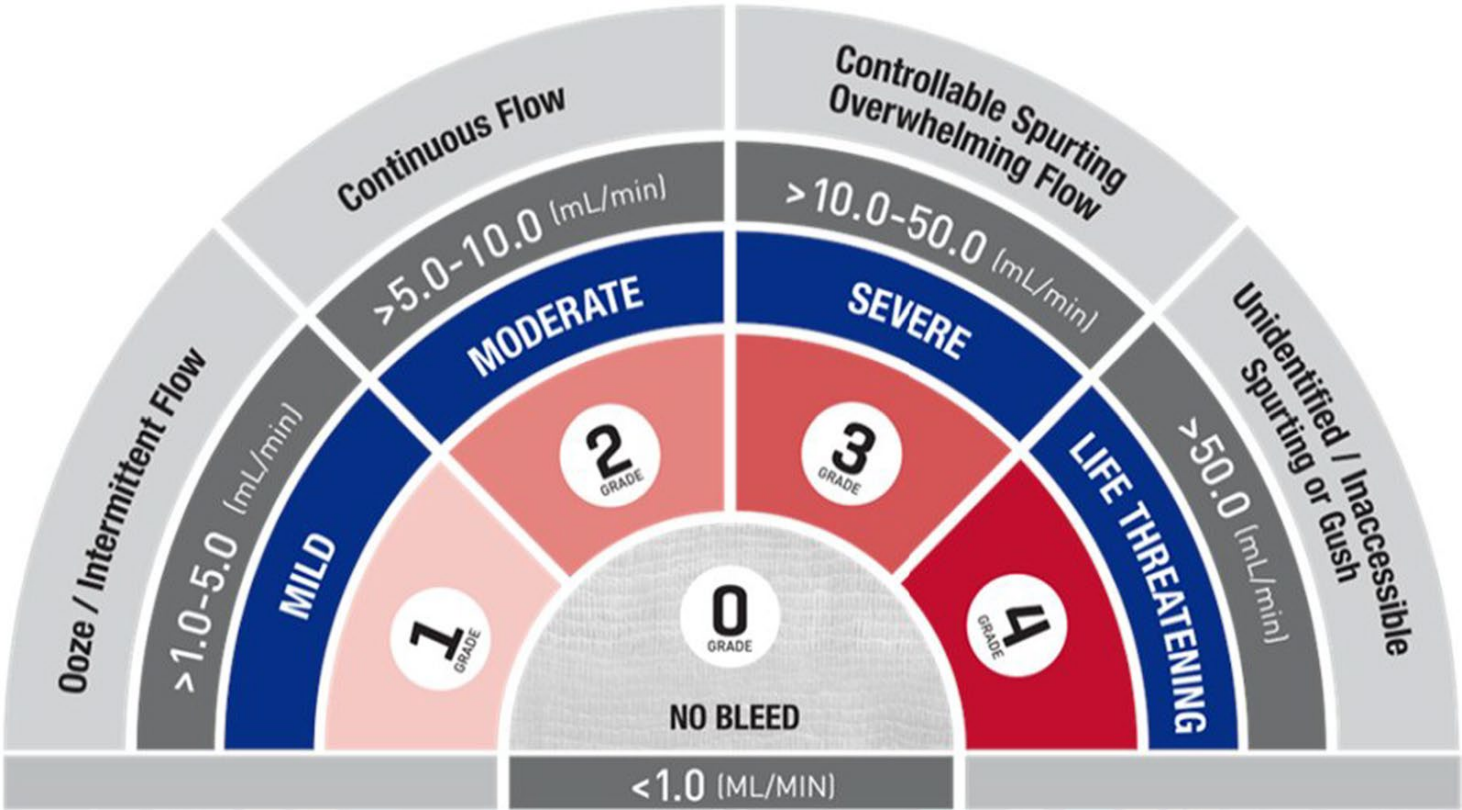
Validated intraoperative bleeding scale grading. Validated intraoperative bleeding (VIBe) scale grades are based on visual field and anatomic appearance, qualitative description, and visual estimated blood loss. Grade 0: No bleeding; reflects clinically insignificant and unremarkable bleeds. Grade 1: Mild; bleeds represent a general ooze, which well up over 1–2 min after blotting with gauze. Grade 2: Moderate; bleeds visibly well up after blotting, and are usually considered distracting to the surgical procedure. Grade 3: Severe; rupture of venous plexus during posterior lumbar laminectomy. Grade 3 bleeds well up immediately after blotting, and require treatment to continue with the surgery. Grade 4: Life threatening; Grade 4 bleeds are life-threatening and require immediate surgical treatment *Source*: VIBe Scale [Internet] [43]

The validated intraoperative bleeding scale (VIBe) facilitates the accurate and reproducible description of bleeding during surgery (Fig. 1) [42]. The surgeon grades intra-operative bleeding according to a five-point validated bleeding severity scale based on the visual and anatomical appearances, the qualitative description, and the estimated rate of blood loss. The scale is a Likert-type scale in which the surgeon assigns a grade based on the general agreement of the bleeding descriptors. The scale was developed and validated amongst surgeons from different surgical specialties, including cardiac, thoracic, and vascular surgery, according to the Food and Drug Administration guidelines for validation of a clinician-reported scale [42]. This surgeon-validated scale shows excellent reliability for the description of different intra-operative bleeding scenarios between surgeons.

The VIBe scale was principally developed for use in clinical trials comparing the efficacy of different haemostatic agents, but the major clinical benefit is a more accurate and reproducible description of intra-operative bleeding. The VIBe grades remove the inherent subjectivity of the assessment of intra-operative bleeding and introduce a common language to describe bleeding for surgeons, anaesthetists, and theatre scrub staff. This shared understanding supports the more effective management of intra-operative bleeding for a patient, particularly the choice of the most appropriate haemostatic agent for the grade of bleeding for rapid and consistent haemostasis [44]. The use of the scale has the potential for reduced peri-operative blood loss, less waste and more effective use of haemostatic adjuncts, reduced need for blood and blood product transfusion, and overall improved patient outcomes. Modelling by Baxter (Fremont, CA, USA), suggests that the introduction of the VIBe scale in cardiac, thoracic, and vascular surgery would have a beneficial economic impact due to the optimal selection of haemostatic agents, reduced intra- and post-operative bleeding, and reduced transfusion costs [45].

The VIBe scale has been adopted across multiple surgical specialties and preliminary feedback from surgeons is favourable highlighting the advantages of a common language to describe intra-operative bleeding and to guide the most effective haemostasis.

### Adjuncts and surgical haemostats

#### Types and indications

Surgical haemostats are adjuncts whose purpose is to complement and consolidate surgical techniques including ligatures and other standard procedures for haemostasis. Haemostatic agents are absorbable, topical agents used as adjunctive therapy for surgical bleeding sites that are uncontrollable by suturing or conventional methods of haemostasis [46]. There are a number of topical haemostatic agents currently available for use in surgery. They can be broadly divided into two categories, passive or active, based on their mechanism of action, along with tissue sealants [47] (Table 1). Based on their profile, their indication, efficacy and their cost differs. The choice of a haemostat depends on the anticipated bleeding risk, ability to achieve haemostasis with conventional surgical techniques, the need for a bolstering effect at the site of bleeding, and the degree of derangement of the coagulation system. While passive haemostats are dependent on a patient’s own clotting cascade to achieve durable haemostasis, active haemostats are effective in patients with deranged coagulation by providing extraneous fibrinogen and thrombin, thus playing a proactive role in clot generation [48].

**Table 1:** Types and Indications of Surgical Haemostats and Tissue Sealants

	Passive	Active	Tissue sealants
Types	Oxidised cellulose (regenerated) Collagens Powders Gelatin sponges Polysaccharide spheres	Flowables (thrombin and gelatin) Fibrin sealants Advanced patches (with fibrin sealants or polyethylene glycol)	Fibrin based Synthetic (i.e. cyanoacrylates or polyethylene glycol) Semi-synthetic (i.e. glutaraldehyde–albumin)
Mode of action	Contact activation and platelet aggregation Relies on the patient’s ability to generate clotting factors	Functions independently of the patient’s ability to generate clotting factors	Functions independently of the patient’s ability to generate clotting factors
Bleeding range	Mild (capillary oozing) to moderate bleeding	Broad range of active bleeding	Pre-emptive for anticipated bleeding
Patient factors	Intact coagulation Limited effect in heparinised and/or anticoagulated treated patients	Compromised coagulation Effective in heparinised and/or anticoagulated treated patients	Compromised coagulation Effective in heparinised and/or anticoagulated treated patients

Passive haemostatic agents, such as collagen, cellulose, gelatin, and polysaccharide spheres act passively through contact activation and by providing a structure to support platelet aggregation and clot formation [47, 49, 50]. Passive non-flowable haemostats are often used to address bleeding ranging from mild capillary oozing to more broad moderate bleeding [50]. As the mechanism of action of passive agents is dependent on a functioning coagulation cascade, the efficacy of passive agents is reduced in patients treated with anticoagulant or antiplatelet medications or with other coagulation disorders [51, 52].

Active flowable haemostatic agents (i.e. thrombin with gelatin), fibrin sealants, or advanced patches promote haemostasis directly by acting at the end of the coagulation cascade [47, 52]. Active agents accelerate natural clot formation process, are effective regardless of whether patients have been treated with anticoagulant or antiplatelet medications, and function independently of the patient’s ability to generate clotting factors [47, 49, 50]. Active flowable haemostatic agents are advantageous in surgery because they can conform to wound contours and fill deep lesions [50]. Unlike passive haemostats, active haemostats can control a wider range of bleeding grades [47, 50].

Topical sealants may be fibrin-based, synthetic (cyanoacrylates or polyethylene glycol), or semi-synthetic (glutaraldehyde–albumin) [49].

#### Strategies and application

The European Association for Cardio-Thoracic Surgery (2017) guidelines recommend considering sealants in situations where conventional approaches to surgical and medical improvement of haemostasis are insufficient and where bleeding is more localised.

The basic principles and most effective strategies to employ haemostats can be broadly defined as:

I. Pre-emptive Application of Sealants

Fibrin sealants are generally composed of a combination of frozen fibrinogen and thrombin that are mixed to produce a liquid flowable form during application that generates a stable fibrin clot similar to the end stage of the natural coagulation cascade. When the bleeding risk is high, application of a sealant before the vessel is de-clamped or blood flow restored allows for pre-emptive sealing of potential bleeding sites. The use of a sealant protects the surgical site and minimises bleeding from needle holes or vascular tissue. Synthetic sealants, commonly known as glues, are composed of cyanoacrylate and polyethylene glycol (PEG) or albumin-glutaraldehyde compounds. An advantage of the synthetic sealants is that they do not require thawing before use. However, they produce a much firmer material which is of benefit for chamber or vessel wall reconstruction, but which may be associated with certain side effects [53].

II. Pro-thrombotic Use of Gelatin and Thrombin Matrix

These active haemostats are indicated in the presence of blood which is absorbed by the thrombin-coated gelatin granules causing them to swell within the confines of the application zone. The thrombin reacts with the patient’s fibrinogen to form a more stable clot achieving haemostasis directly at the bleeding sites [54]. To achieve a tamponade effect by physically stopping blood flow, the use of haemostats in the form of patches or sheets serve to provide a surface that allows blood to clot rapidly. These buttress products may contain active components to complete the coagulation cascade. For example, there is a two-layer sponge with one side comprised of collagen and the other of fibrinogen and thrombin, polyethylene glycol, and oxidised cellulose, or are devoid of them (e.g., collagen or oxidised regenerated cellulose) [55]. Their application may be combined with the liquid sealants or flowable matrix, to create a barrier to stop bleeding and stabilise the clot.

The strategy of preventing bleeding by employing a sealant followed by the use of a matrix to generate clot formation at puncture bleeding sites maximises the efficacy of these different haemostats and minimises their waste. The COVID-19 pandemic has made a significant impact on various supply chains through finance, lead time, demand changes and production performance, although specific supply disruptions to haemostatic agents have not been reported [56].

#### Preparation and nursing perspectives

The World Health Organization (WHO) Surgical Safety Checklist, introduced in 2008 (with amendments by the National Patient Safety Agency in 2010), created the “five steps for safer surgery”; team briefing, patient sign-in, timeout, sign-out, and debriefing, which have become standardised safety checks in operating theatres across the UK [57, 58]. Incorporating multi-disciplinary team members, learning opportunities involving communication, perceived authority gradients, situational awareness and psychological safety have been brought to the forefront to decrease errors, and avoid adverse events [59].

A cardiothoracic theatre scrub nurse’s role in patient safety in the UK involves the knowledgeable and safe preparation of the theatre, and their active participation in an effective team brief enables this to occur. The team brief which is often led by the consultant surgeon includes the discussion of any expected and unexpected events, enabling emergency and stand-by items to be discussed in a calm and non-confrontational environment, allowing timely preparation, and avoiding preventable delays in treatment during the procedure. During the COVID-19 pandemic, changes to UK hospital Infection Prevention and Control protocols, alterations to the physical set-up of theatres and availability of consumables inside a theatre may contribute to additional delays in preparation, including the opening of surgical consumables and thawing of haemostats. An effective team briefing can eliminate these additional theatre nursing blood management concerns.

The Nursing and Midwifery Council code identifies continued professional development as a requirement to maintain competence and further develop clinical skills [60]. During the COVID-19 pandemic, delivery and access to training across the UK have been altered due to restrictions in physical access across healthcare settings. Miech et al. [61] highlighting the role of clinical champions as important positive influences on implementation and effectiveness. During the COVID-19 pandemic, nurse champions for product-specific equipment and consumables training, including haemostats within theatres, have facilitated training to continue in situ. Utilising a combination of virtual company support, demonstration kits, and in-house equipment, training can be maintained within the theatre and during clinical governance sessions. Maintenance of training within the nursing team and designation of haemostatic champions are positive factors in a theatre nurses’ ability to support the multidisciplinary team in surgical blood management options.

## Specificities of thoracic and robotic surgery

As with all forms of surgery, bleeding can occur in a number of scenarios during thoracic surgery:

I. Bleeding in relation to approach be it open thoracotomy or minimal access surgery – Video-Assisted Thoracoscopic Surgery (VATS) or Robotic-Assisted Thoracic Surgery (RATS)

Prevention is often better than cure when it comes to management of bleeding in relation to wounds. Despite planned meticulous haemostasis, bleeding from intercostal vessels or adhesions is possible. Electrocautery and/or haemostatic clips are usually sufficient to control these. At the posterior end of a thoracotomy wound (especially where a portion of rib has been excised), other adjuncts such as oxidised cellulose or active flowable haemostat (gelatin & thrombin) can be used [54, 62]. The use of VATS and RATS in Thoracic Surgery has increased [63, 64]. Use of electrocautery for adequate haemostasis is important particularly in robotics, where the ports are tight with the movement of the arms. This minimises the frustration of constant telescope changes due to impaired vision and also prevents insidious intraoperative and postoperative blood loss.

II. Bleeding from lung parenchyma

Any procedure involving dividing lung tissue may result in bleeding and air leak. These two complications are inextricably linked. Again, prevention is better than cure, and the method of parenchymal division is important in minimising blood loss from the lung. Good quality appropriately-sized staplers, with or without buttressing, can minimise bleeding. There is some evidence that powered staplers (especially robotic) are associated with less blood loss and fewer air leaks [65]. Other adjuncts that can be applied to the staple line may further reduce bleeding risk. Fibrin sealants are effective and have the potential added advantage of reducing air leaks [66, 67]. Polyethylene glycol products are used, but like tissue patch products, these are predominantly for the prevention or management of air leaks [68–70]. Synthetic sealant (i.e. cyanoacrylates) is an effective haemostat in cardiac surgery, and has been used in thoracic surgery for haemostasis as well as prevention of air leaks [71–73].

III. Bleeding from major vessels

Anatomical lung resection for the treatment of primary lung cancer is perhaps the single most common procedure in thoracic surgery [74, 75]. Dissection of the main hilar structures (bronchus, vein and artery) is an integral part of these procedures. Although a low pressure system, the pulmonary artery is an unforgiving structure: avoidance of damage is vital. When bleeding does occur, accurate closure by clip or suture is required [76]. Regardless of approach, if continued bleeding occurs, active haemostatic agents can be useful [77, 78]. Catastrophic bleeding from the pulmonary artery in RATS is an ever-present risk [79]. An integral component of surgeon and robotic team training is preparation for this rare but important complication. This involves development of standard operating procedures (SOPs) for crisis resource management.

## COVID-19 considerations for haemostasis

At the time of writing, the number of confirmed cases of COVID-19 worldwide is approaching 614 million. In the UK, the number of confirmed cases has surpassed 23 million and there have been more than 207,200 deaths [80]. The number of operations fell 34% in 2020/21. Adult surgery activity reduced by 80% and 60% during the two peaks of coronavirus disease (COVID-19) admissions. Around 10,000 patients across the UK did not have the heart surgery they should have done (National Cardiac Audit Programme (NCAP) [81] Report). Nevertheless, the implementation of specific SOPs such as testing, isolation, and consequent use of personal protective equipment (PPE) allowed the development of a system for safe surgery. A propensity-matched study of outcomes at 6 months comparing cardiac surgery during and prior to the COVID-19 pandemic finding no differences in 30-day all-cause mortality, 6-month survival, and length of post-operative stay [82]. However, further study of 773 patients confirmed an increased risk of early and late bleeding complications in COVID-19 patients undergoing cardiac surgery [83].

The COVID-19 pandemic is the defining global health crisis of our time and the greatest challenge of our generation. Government restrictions on movement and activity to reduce the spread of COVID-19 have affected all aspects of the daily activities of the general population. Lifestyle and diet have been dramatically altered, often through fear and confinement. A large percentage of national populations have adopted working from home routines that may be more sedentary, with poorer diets, and deficiency of nutrients, including sources of vitamins, folic acid, and iron. This has increased the population’s vulnerability to COVID-19 with correlated coagulopathy [84, 85]. With reduced travel, populations in northern hemisphere countries may become vitamin D deficient as a result of reduced exposure to sunlight [86]. Pandemic restrictions and social distancing have reduced access to blood donation centres. There was a significant shortage of blood supplies worldwide, and encouraging healthy donors to donate blood has proven to be a challenge [2].

As the pandemic evolved, we expected to see an increased number of late presentations of myocardial infarction with mechanical complications, such as ventricular septal and papillary muscle rupture, but the rates of admission to UK hospitals for these feared complications were similar to those of previous years. However, national audit statistics revealed a higher than average mortality for non-COVID diagnoses during surges of the pandemic, suggesting that some patients were dying at home because of an understandable reluctance to seek medical attention because of concerns over the transmissibility of COVID-19 [87]. With only 10.5 critical care beds per 100,000 inhabitants (compared to 33.9 in Germany and 25.8 in the USA), the UK was severely challenged for ICU capacity during the pandemic [88]. Cardiac surgery was limited to emergencies and urgent cases [89]. Acute coronary syndrome patients receiving dual antiplatelet therapy who need emergent or urgent cardiac surgery are known to be at higher risk of severe bleeding. The use of intraoperative active adsorbers for patients receiving Ticagrelor addresses this issue improving peri- and post-operative outcomes [90]. The general necessity to reduce ICU stays to a minimum resulted in an even more need for meticulous haemostasis during cardiothoracic surgery. Haemostatics generally used to treat active bleeding gained new relevance when applying them prophylactically to reduce the number of bleeding complications and returns to theatre [91].

## Conclusions

Bleeding remains an important complication of cardiothoracic surgery with a pejorative impact on outcome and resources. This was further amplified during the COVID-19 pandemic. Patient blood management in the UK is a multidisciplinary process that starts in parallel with the patient’s referral to surgery. We outline an integrated approach that aims to identify, address and adapt a blood preservation strategy. We refer to a validated bleeding scale to enhance communication and identify optimal employment of passive and active haemostats as adjuncts when conventional surgical techniques fail to achieve haemostasis.

## Data Availability

Not applicable.
